# Eye Strain on the Nervous System*Read at the County Medical Society meeting and School Teachers’ Institute in October, 1911.

**Published:** 1911-12

**Authors:** G. B. Taylor

**Affiliations:** Cameron, Texas


THE
### TEXAS MEDICAL JOURNAL.
Established July, 1885

F. E. DANIEL, M. D., - - - - Editor, Publisher and Proprietor

Published Monthly.—Subscription, $1.00 a Year.

VOL. XXVII. AUSTIN, DECEMBER, 1911. No. 6.

The publisher is not responsible for the views of the contributors.

### Original Articles.

For Texas Medical Journal.

### Eye Strain on the Nervous System.*

BY G. B. TAYLOR, M. D., CAMERON, TEXAS.

The most constant condition caused by eye strain is, of course, the headache. This headache may develop slowly or rapidly, may be centered in one eye, one side of the forehead, or one side of the head, or may be referred to both eyes. In fact, there is no part of the head that may not ache from eye strain. Very frequently, however, one eye is more likely to be affected than the other, as one eye is likely to be unlike the other eye, and be more defective than the other eye.

Astigmatism are the most frequent causes of eye-strain headache. The pain is perhaps more frequent in the supra-orbital region, but is often in the temple, and may be frequently referred to the inner angle of the eye, especially in astigmatism. It perhaps occurs very frequently in this region on account of the overactivity of the superior oblique muscle, which endeavors to overcome an astigmatic affection. Headaches from defective vision from any reason, and especially when a person becomes presbyopic and has not glasses to correct it, are more likely to be in the occipital region. Such headaches more frequently occur in the early morning and are discovered by the patient on awakening.

*Read at the County Medical Society meeting and School Teachers' Institute in October, 1911.

Children are likely to have headaches begin at any age, but perhaps more frequently after one or two years of school work. These headaches are likely to come periodically, perhaps once a week, perhaps once in two weks, perhaps only once a month, but with a constant tendency to become more frequent, little by little. They sooner or later become a migraine, which is typically a headache for a number of hours, followed by nausea, vertigo, vomiting, prostration, sleep and recovery. The title given to most of these headaches by the laity is a bilious attack. He then attributes his trouble to his liver, or finds serious fault with his stomach. If he is constipated, he lays it to that, as he finds following a brisk purgative the headache disappears. He, therefore, thinks it due to constipation.

Girls and women with these eye defects are more likely to have headaches before or during menstruation, and they attribute to that function. Some soon learn to become suspicious of their eyes on account of having the headache after theater-going, card playing, shopping, sewing or reading too long, or, if they are office clerks, after an extra amount of proof reading, or mathematical work. That eye defects with their headaches are on the increase is perfectly logical from the over use of the eyes in the civilized community of today.

The young child, instead of playing by itself, is shown figures, pictures, objects to put together, and finally comes the ill-judged kindergarten eye proposition. This is a distinctly injurious custom. Very young children should be discouraged from any near work, and in day nurseries and primary schools the most of the teaching requiring visual labor should be by large pictures and charts hung at some distance from the pupil's eyes. If a child has red eyes, holds its book close, complains of not being able to see at a distance, looks at objects sideways, or between partially closed lids, or squints or complains of headache, browache or pains in the eyes, it is the parent's or teacher's duty to send it to a competent oculist. If the oculist decides that glasses are necessary, they should be put on at once in spite of any foolish prejudice, for they will save and promote the physical and intellectual development of the child and prevent many years of suffering and perhaps irreparable ocular disease. Young children with defective vision are often called stupid and unattentive, and actually punished for physical faults, which should have been detected and remedied long before.

We should also note the greater number of hours that even children, to say nothing of grown people, are up and alert with eyes never at rest, as compared with those which our grand parents spent in that way.

I am going one step further—more eyes must have glasses, and more young eyes must have glasses, and the glasses must be better than they ever were, and those who need bifocal glasses must have them ground into one lens, so as to have no disturbance whatsoever of almost perpetual vision. Like everything else in life, the supply must meet the demand, and one has but to count the ever-increasing number of opticians to realize that the world needs glasses.

There is a class of persons calling themselves scientific opticians, refracting opticians, ophthalmistic, and the like, who, without even serving the apprenticeship of a skilled optician, without the slightest medical training and with very little or no mechanical knowledge, undertake to prescribe glasses, as well as to sell them. They advertise—eyes examined free, and sometimes travel rapidly from town to town, calling themselves Doctor or Professor. It is hardly necessary to reiterate that it is dangerous to entrust the care of such a delicate and intricate organ as the eye to a non-medical person. This is the business of the oculist, who is an educated physician, and has had long experience in medical colleges, clinics and hospitals, and who is devoting his life to the study of his profession.

Can anything short of a competent medical education enable any man to differentiate and diagnose as these symptoms appear in the eye? Such conditions as albuminuria, diabetes, arteriosclerosis, brain tumor, accessory, sinus disease, toxic amblyopias, syphilis, tubercle, and so on, to say nothing of the dangerous localized intra-ocular diseases. Unfortunately, the world does not realize the delicateness and the wonderful constitution of its eyes. Therefore, a larger part of the world thinks that any optician traveling, or otherwise, can fit eyes to suitable glasses. All the patient is asked to decide is whether he sees better or worse with this or that lens. Undoubtedly an enormous amount of serious damage is done to the eyes by ignorant opticians.

I do not know of any class of medical work that requires more patience and more good judgment than the decision by an oculist as to just what lens a person with defective eyes requires. In the first place, the patient is more or less unable to determine accurately when or how he sees the best; in the next place, when the eye is made as immovable as possible, with mydriatics or cycloplegics, still there is sufficient mobility to impair accurate judgment as to just what sort of optical instrument the eye is.

One fact should be impressed on the patient, and that is that if an oculist tells him he has astigmatism, the glasses must be frequently adjusted. They must be absolutely straight to correct the meridian of the eye; that is, abnormal. Another fact that should be impressed on the patient is that a small defect, sometimes so slight that the patient feels that he does not need lenses, may cause more reflex trouble than a serious defect. Also, the amount of trouble that will be caused by an eye defect depends on the individual—nervous, high-tension or neurotic patients have much more reflex trouble than do more phlegmatic individuals, or those who may not be doing high-tension work.

All physicians now, more or less, recognize recurrent headaches as due to eye strain, but a larger number do not recognize that the patient may have stomach and heart reflexes without headaches, and still due to eye defects. Dizziness, gastric indigestion, even nausea or vomiting, may occur without any headache whatever, and still be due to eye strain. Cold hands and feet, chilly sensations, faint feelings, palpitation and irregular heart and pain referred to the cardiac region, so as to cause the patient to believe he has heart disease, may be due to eye strain.

If the patient comes to the physician with a headache, or wants to know what he shall do when he has his next headache, what shall we give him? It is often unwise to give him a prescription. It is better to give the patient some tablet from the office or from the pocket case, made according to a formula, which is satisfactory to the physician. It is bad to adopt any opium treatment for these headaches; on the other hand, one is often driven by the very intensity of the condition to the limit of medical resources, and sometimes cardiac depression is so serious that it becomes a question of either morphine or larger doses of alcohol. Patients who have these terrible migraine attacks frequently can not well stand coal-tar products, so often repeated, as unless the dose is enormous, the results of their administration is nil. A morphine habit and an alcohol habit, to say nothing of the frequent acetanilid habit or caffeine habit, may be acquired on account of eye strain.

---

For Irrigating Membranes.—Sixteen grains of salicylic acid and 96 grains of boric acid dissolved in a pint of sterile water makes Thiersh's Fluid. This is useful for cleansing serous and mucous membranes, such as those of the mouth and eye, and it may be used for any purpose where irrigation is employed for cleansing purposes.—*Medical Brief*.

